# Neural correlates underpinning synchronized movement of double scull rowers

**DOI:** 10.1038/s41598-021-82392-0

**Published:** 2021-02-03

**Authors:** Takahiro Hirao, Hiroaki Masaki

**Affiliations:** Faculty of Sport Sciences, Waseda University, 2-579-15 Mikajima, Tokorozawa, Saitama 359-1192 Japan

**Keywords:** Empathy, Human behaviour

## Abstract

We investigated the neural correlates underpinning synchronized movement in rowers using a neural index for social interaction termed the phi complex. Phi 1 and phi 2 indicate the enhancement and reduction of mirror neuron activation, respectively. We hypothesized that in a leader–follower relation, followers would exhibit a larger phi 2 power than leaders due to enhanced mirror neuron activation by the followers to accurately mimic their partner’s movements. We also expected that brain activation underlying social interaction would be enhanced during synchronization. Although phi 2 was not modulated by role (leader vs. follower) or condition (usual-pair vs. unusual-pair), the statistical analysis suggested the relationship between the magnitude of phi 2 and empathetic ability in followers in the usual-pair condition. Given that the activation of the mirror neuron system underlies empathic ability, it is plausible that the participants used the mirror neuron system to follow the movement of a usual partner. In other words, the leader in the synchronization did not need to use the mirror neuron system, which was consistent with the result of a larger phi 1 for leading than following the movement. These results suggest that the neural correlates of empathy may be used to synchronize with partners as the follower.

## Introduction

In various sporting competitions, superior performance includes highly synchronized movements among athletes. For example, in the Olympic games, artistic swimmers are required to act simultaneously and synchronize with their partner’s actions precisely. Rowing also requires a high synchronicity to drive a boat fast and efficiently, as do athletes. Using two oars, rowers in double sculls aim to reduce boat fluctuation, and this leads to more effective movements through movement synchronization^1^. Previous studies have focused on the biomechanical aspects of boat rowing, such as oar peak force, duration of stroke, rowing angle, and inter-stroke interval^1–4^. However, the ability of social interaction among rowers was not considered as a factor that enhances rowing performance in these studies.

Human beings form social groups and acquire fundamental social skills to achieve interpersonal interactions (e.g., non-verbal communication) that comprise mutual information exchange among people and facilitate the synchrony of interpersonal unintentional motor coordination using visual information^5^. Thus, information exchange between two rowers may be critical to achieve movement synchronization in double scull rowing.

Research in social neuroscience has identified the mirror neuron system (MNS), which consists of the inferior frontal gyrus and the inferior parietal lobule^6^. Studies have reported that the MNS is involved in social interaction^7,8^ because it is activated when individuals interact with others, encompass empathy, imitate movements, and understand the action intention of others^9–14^. Further, the activity of the right inferior frontal area in the MNS is correlated with empathy ability^14^. Previous studies have reported that the Interpersonal Reactivity Index (IRI) is a useful questionnaire that evaluates the cognitive aspect of empathy, which is correlated with activation of the MNS^14–16^. Recent neural imaging studies have begun to elucidate the neural basis of the MNS, and a meta-analysis that included 70 functional magnetic resonance imaging (fMRI) studies revealed that the right inferior parietal cortex played a role in social interaction, such as empathy^17^. Both frontal and parietal regions, including the right inferior parietal lobule, are crucial for imitation behaviours in humans^18^.

Although fMRI has allowed for the identification of brain regions that underpin social interactions with high spatial resolution, movements of participants are exceedingly restricted in the fMRI scanner; thus, fMRI recordings are not suitable for observing neural activities underlying the dynamic coordinated movements of athletes. Tognoli and Kelso (2015) reviewed the neural indices of dyadic social interactions using electroencephalogram (EEG) recordings^19^. They argued that among several neural indices of social interaction, the phi complex that emerged over the centro-parietal regions was a useful neuromarker of dyadic social interaction with which mutual information exchange occurred during coordinated and uncoordinated movements^20^. The phi complex consists of two frequency ranges: phi 1 (approximately 10–12 Hz) and phi 2 (approximately 12–13 Hz)^20^. Powers in these frequency ranges are asymmetrical, which is consistent with fMRI findings^17,18^. The phi complex emerges when the powers of these frequency ranges in the right hemisphere are subtracted from those in the left hemisphere. In a study conducted by Tognoli et al. (2007)^20^, pairs of participants were instructed to sit face-to-face and perform self-paced rhythmic finger movements. Behavioural analysis classified the finger movements into synchronized and unsynchronized movements. The powers of phi 1 and phi 2 over the centro-parietal regions were increased for unsynchronized and synchronized movements, respectively. Given that the topographical distribution of the phi complex corresponds with the anatomical regions of the MNS^21^, phi 1 may reflect the inhibition of the MNS and phi 2 may reflect activation of the MNS^20^.

Considering the functional significance of the MNS underpinning social interactions^9–14^, it is plausible that the MNS may also contribute to highly synchronized movements in boating. If rowers have acquired skills of unintentional motor coordination through their boating experience, brain activities associated with social interactions can be modulated by coupling a pair of rowers to coordinate movements because rowers in double scull are required to share their attention with specific partners. In a hyperscanning fMRI study^22^, the brain activities of pairs of subjects in their first interaction as a dyad were scanned before and after the execution of a joint attention task that required one of the participants to chase a gazing point of their partners. They found that the inter-individual neural synchronization in the inferior frontal gyrus (i.e., a part of the MNS) was increased after the experience of shared attention; therefore, the experience of shared attention impacted the neural response when individuals were gazing at each other^22^. Based on previous findings, we presumed that the neural activities observed during double scull in usual rowing partners who play and practice in the boat race would differ from those observed in unusual rowing partners when the rowers play.

As such, this study sought to elucidate the neural dynamics that underpin interpersonal movement synchronization of double scull pairs using EEG hyperscanning. We assessed the neural correlates of boat rowers using a simple coordination task that was designed to test a leader–follower relationship. In the coordination task, dyadic pairs were asked to flex and extend their wrist with and without synchronizing their movement (i.e., the sync and control conditions). When synchronizing their movement, one participant was asked to lead the movement and the other followed the movement of the leader.

To investigate the neural activities associated with synchronization in double scull pairs, we compared neural activations for the social interaction (i.e., phi complex) during synchronized movements between the usual- and the unusual-pair condition. In the usual-pair condition, a participant performed the simple coordination task with the rower whom he/she had collaborated with in double scull boat competitions. In the unusual-pair condition, the participant executed the task with another rower whom he/she had not collaborated with in competitions. Comparing neural activities during the usual-pair condition with those during the unusual-pair condition would reveal neural correlates underpinning the coordination of double scull boating.

We calculated the phi complex as an index of MNS activation during wrist movements. We hypothesized that a larger phi 2 (more activation of the MNS) would be observed when the participant followed the leader’s movements than when the participant was the leader of the task movement because the follower would more strongly activate his/her MNS to mimic their partner’s movements. Conversely, we hypothesized that a larger phi 1 would be observed during leading movements than during following movements because the leader would be required to inhibit the MNS to ignore the partner’s movements. Finally, we hypothesized that brain activations underpinning social interaction would be enhanced when the original double scull partners interacted with each other because they had established an interpersonal social connection through the experience of shared attention during double scull rowing.

## Results

### Wrist movements

**Figure 1:**
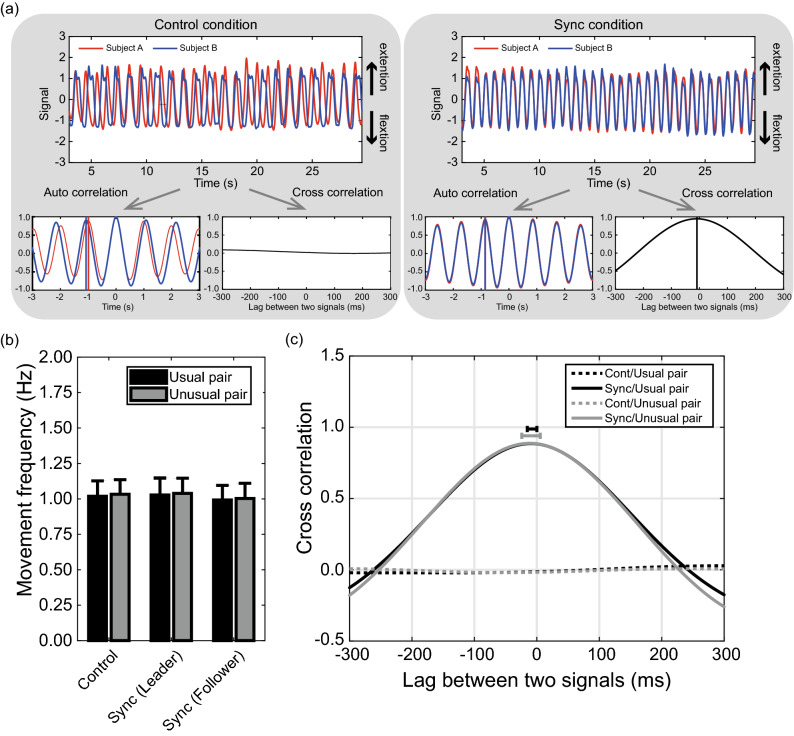
Behavioral results. (**a**) Typical example of wrist movement data. For each condition, the normalized wrist movement data and the results of the auto correlation and cross correlation in a representative trial of a pair are shown. In the results of the auto correlation, a vertical solid line indicates the first peak latency of the auto correlation curve. In the results of the cross-correlation, the vertical solid line indicates the latency of the peak value of the cross-correlation curve. An explicit peak of the cross-correlation curve was not observed in the base condition because the wrist movements of two participants were not temporally synchronized. Therefore, the solid vertical line was not depicted on the cross-correlation curve in the base condition. (**b**) Wrist movement frequency was identified by the first peak of an auto correlation curve. The error bars in the bar graph represent standard errors. (**c**) Grand-averaged cross-correlation curve. The positive value of the lag indicates that the leader led the movements of the follower; contrarily, a negative value of the lag indicates that a follower led the leader’s wrist movement. In sync trials, standard deviations for a time lag of the peak of curves are represented as horizontal error bars.

Figure 1b shows wrist movement frequencies of participants in each condition. A two-way ANOVA confirmed that there was neither a main effect of trials (control, sync-leader, and sync-follower trials) [*F*(3, 45) = 4.46, *p* = 0.02, η_p_^2^ = 0.23] nor of conditions (usual- and unusual-pair condition) [*F*(3, 45) = 4.46, *p* = 0.02, η_p_^2^ = 0.23] on movement frequency.

Grand-averaged cross-correlation curves are shown in Fig. 1c. The cross-correlation between two signals that represent a transition of the angle of wrist movements in dyadic pairs were calculated in each condition. The cross-correlation curves were mountain-shaped in the synchronized trial, symmetrical about the coordinate origin (i.e., the peaks were − 7.7 ms and − 9.3 ms in the usual- and unusual-pair conditions, respectively), whereas they were flat-lined in the control condition, with zero correlation values. The peak time of the cross-correlation curve on the sync trial in each condition was compared with 0 ms using a one-sample *t*-test. The peak times in the usual- and unusual-pair conditions were not significantly different from 0 [*t*(8) = 1.68, *p* = 0.13, *d* = 0.79; *t*(9) = 1.98, *p* = 0.08, *d* = 0.89]. These behavioral results indicated that the synchronization of the wrist movement was not affected by condition (usual- and unusual-pair condition).

### Phi complex

**Figure 2:**
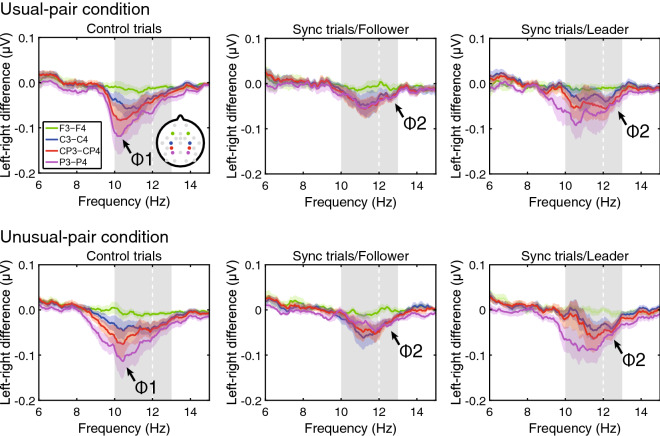
Results of the frequency analysis for the phi complex. The difference in amplitudes for each frequency between the left hemisphere and the right hemisphere was calculated as described in a previous study^20^. The grand means of all 16 participants are plotted in the figure as solid lines. Standard errors are overlapped with the line plots as shaded areas. Grey shaded areas represent scored areas for the phi complex.

Figure 2 shows plots of amplitude differences between the left and right hemispheres. Consistent with Tognoli et al.^20^, the spectral component in a phi-complex range was cancelled out due to the asymmetrical magnitude between the left and right hemispheres over the frontal region. Conversely, larger amplitudes were observed in the right hemisphere than those in the left hemisphere over the central, centro-parietal, and parietal regions. In this study, to assess the neural correlate under the motor coordination of athletes, roles in the motor coordination and pairing were used as factors in ANOVAs.

A three-way ANOVA with repeated measures on the variables of trial (sync-leader/sync-follower/control trials), condition (usual-/ unusual-pair condition), and region (frontal/central/centroparietal/parietal area) revealed a main effect of region on phi 1 [*F*(3, 45) = 6.15, *p* = 0.008, ε = 0.60, η_p_^2^ = 0.29]. The phi 1 in the frontal area was smaller than that in the parietal area [*t*(15) = 3.19, *p* = 0.037, *d* = 1.08]. There was also a significant interaction between Region and Trial on phi 1 [*F*(6, 90) = 2.92, *p* = 0.039, ε = 0.55, η_p_^2^ = 0.16]. Post-hoc comparisons showed that the magnitudes of phi 1 in the central and parietal area tended to be larger than those in the frontal area in the control trial [*t*(15) = 2.70, *p* = 0.098, *d* = 0.79, *t*(15) = 2.82, *p* = 0.078, *d* = 1.01, respectively]. Moreover, the magnitudes of phi 1 in the parietal area tended to be larger than those in the frontal area in the sync-leader trial [*t*(15) = 2.77, *p* = 0.086, *d* = 0.87]. No other significant differences were observed in the post-hoc comparisons [*ts*(15) ≤ 2.69, *ps* ≥ 0.010, *ds* ≤ 0.83]. Statistical analyses by the Shapiro–Wilk test showed that some phi 1 data did not have normal distributions (*Ws* ≤ 0.84, *ps* ≤ 0.04). Therefore, the significant differences found in the parametric analysis were re-tested by using the non-parametric analysis. The results of the non-parametric analysis were similar to the results of the parametric analysis. Concretely, the non-parametric analysis confirmed that the phi 1 in the frontal area was smaller than that in the parietal area (*Z* = 3.21, *p* = 0.008). Moreover, the non-parametric analyses showed that the magnitudes of phi 1 in the centro-parietal and parietal area were larger than those in the frontal area in the control trial [*Z* = 2.84, *p* = 0.027; *Z* = 2.90, *p* = 0.023, respectively]. Moreover, the magnitudes of phi 1 in the parietal area tended to be larger than those in the frontal area in the sync-leader trial (*Z* = 2.90, *p* = 0.023).

A three-way ANOVA also revealed a main effect of Region on phi 2 [*F*(3, 45) = 7.13, *p* = 0.001, ε = 0.47, η_p_^2^ = 0.32]. Phi 2 was smaller over the frontal region than over central and parietal regions [*t*(15) = 3.29, *p* = 0.03, *d* = 0.90, *t*(15) = 3.15, *p* = 0.040, *d* = 1.00, respectively]. Consistent with previous studies, the phi complex was not observed in the frontal area^20^.

As previously described, the parametric and non-parametric statistics for the phi 1 and phi 2 showed that there were no significant differences induced by the trial and/or condition factors although there were significant potential differences among electrodes. Contrary to our prediction, we did not find the effect of a role in the movement coordination and pairing on the phi complex. There was a possibility that we did not capture the neural activity like empathy due to the motor-related activities in the phi complex. To remove motor-related neural activities due to wrist movements in the sync trials, we subtracted the value in the control trial from the value in the sync trials. After subtracting the motor-related activities, the Shapiro–Wilk tests confirmed that the data were normally distributed (*Ws* ≥ 0.86, *ps* ≥ 0.27). A three-way ANOVA revealed a significant interaction between Region and Trial on phi 1 [*F*(3, 45) = 4.46, *p* = 0.02, η_p_^2^ = 0.23]. The phi 1 magnitude tended to be larger when participants moved their wrist as a leader than when they moved their wrist as a follower [*t*(16) = 1.80, *p* = 0.092, *d* = 0.48]. No significant difference was noted in a three-way ANOVA for phi 2 (*Fs* ≤ 1.59, *ps* ≥ 0.23, η_p_^2^s ≤ 0.10).

### Correlation between the magnitude of phi 2 and empathy scores

Consistent with previous findings^19,20^, our results revealed that phi 1 and 2 were absent in the frontal area. Therefore, we analysed the correlations of the subtracted phi 1 and 2 (i.e., sync minus control trials) over the central, centro-parietal, and parietal regions, using Pearson correlation analysis.

**Figure 3:**
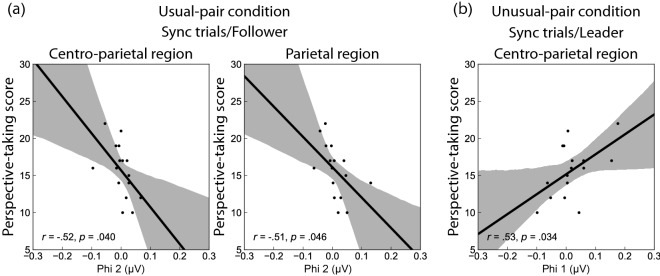
Scatter plots of correlations between changes in the phi complex (from control to sync trials) and PT scores of the IRI. These scatter plots indicate the relation between the individual PT ability and the individual magnitude of the phi complex. (**a**) The two plots represent the correlation between PT scores and phi 2 in followers during movement synchronization in the usual-pair condition. (**b**) The plot represents the correlation between PT scores and phi 1 in leaders during synchronization movements in the unusual-pair condition. Grey shaded areas represent 95% confidence intervals. *IRI* interpersonal reactivity index, *PT* perspective-taking.

Figure 3 depicts scatter diagrams of the correlations between the phi complex and perspective-taking (PT) scores from the IRI. A correlational analysis for phi 1 in each condition revealed that the PT score was positively correlated with phi 1 over the centro-parietal regions when the participant synchronized movement as a leader in the unusual-pair condition (*r* = 0.53, *p* = 0.034). The correlational analysis for phi 2 revealed that empathy was negatively correlated with phi 2 over the centro-parietal regions when the participant synchronized movement as a follower in the usual-pair condition (centro-parietal region: *r* = –0.52, *p* = 0.040; parietal region: *r* = –0.51, *p* = 0.046).

**Figure 4:**
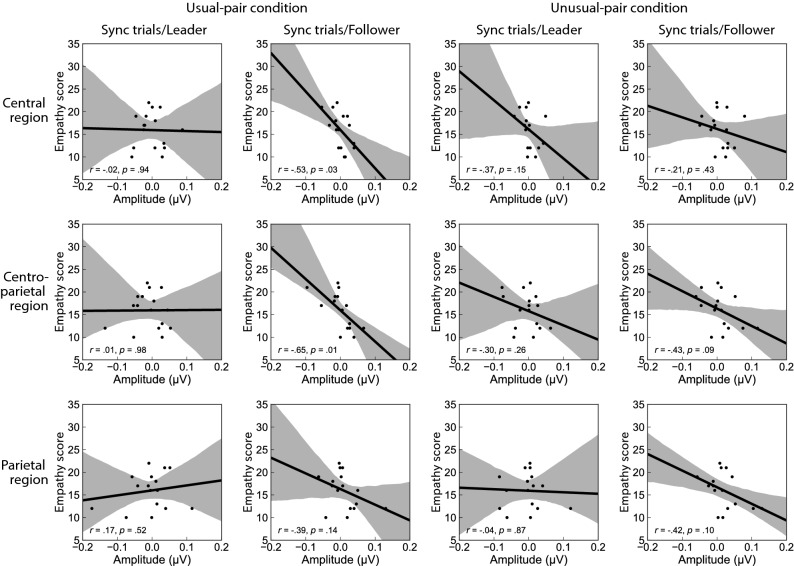
Correlations between changes in phi 2 (from control to sync trials) and EQS empathy scores in each condition. These scatter plots indicate the relation between the individual empathetic ability and the individual magnitude of the phi 2. Grey shaded areas represent 95% confidence intervals. *EQS* emotional intelligence scale.

We also conducted a correlational analysis between the phi complex and emotional intelligence scales (EQS) (Fig. 4). Pearson correlation analysis revealed that phi 2 correlated negatively with empathy scores when participants synchronized their wrist movement as followers. The phi 2 correlated negatively with the empathy scores for the central and centro-parietal regions (*r* = –0.53, *p* = 0.03, *r* = –0.65, *p* = 0.01, respectively), but not for the parietal regions (*r* = –0.39, *p* = 0.14). However, phi 1 was not significantly correlated with empathy (*rs* ≤ –0.49, *ps* ≥ 0.06).

In the Pearson’s correlation analyses, the multiple-correlation correction by a false discovery rate (FDR) approach was performed separately for each item in the questionnaires and each phi component. The results of the correlations before and after the FDR correction are summarized in Table 1. After the FDR correction, the correlation between the phi 2 on the centro-parietal regions in sync/follower trials of the usual-pair condition and the empathy score in EQS was marginally significant, but other correlations were not significant.

**Table 1:** Correlations between the phi complex and the scores of questionnaires. The *r* values of the correlational analyses are shown. Bolded values indicate significance before applying the false discovery rate (FDR) correction. Underlined values indicate the correlation which reached a marginal significance after applying the FDR correction. *p < .05; **p < .001 (in the correlation without the FDR correction). The correlational analyses were conducted using IBM SPSS Statistics version 22 (IBM Corp., NY, USA; https://www.ibm.com/products/spss-statistics).

				EQS	IRI			
				Empathy	PD	EC	PT	FS
Phi 1	Usual pair	Sync/leader	C	− 0.07	− 0.06	0.23	0.05	− 0.10
			CP	0.01	− 0.04	0.18	0.09	− 0.11
			P	0.02	− 0.19	0.01	0.03	− 0.03
		Sync/follower	C	− 0.35	0.08	− 0.22	0.23	− 0.03
			CP	− 0.38	− 0.07	− 0.43	− 0.09	− 0.16
			P	− 0.49	− 0.46	− 0.48	− 0.30	− 0.19
	Unusual pair	Sync/leader	C	0.07	0.07	− 0.09	0.43	0.09
			CP	0.10	0.08	− 0.15	0.53*	-0.04
			P	0.12	0.09	− 0.19	0.07	− 0.26
		Sync/follower	C	− 0.10	0.07	− 0.27	0.34	0.20
			CP	− 0.31	− 0.06	− 0.40	0.11	− 0.01
			P	− 0.41	− 0.20	− 0.42	− 0.01	− 0.21
Phi 2	Usual pair	Sync/leader	C	− 0.02	− 0.20	0.10	− 0.44	− 0.11
			CP	0.01	− 0.08	0.16	− 0.12	− 0.13
			P	0.17	− 0.17	0.17	0.05	− 0.09
		Sync/follower	C	− 0.53*	0.01	− 0.11	− 0.44	0.13
			CP	− 0.65**	0.02	− 0.38	− 0.52*	0.12
			P	− 0.39	− 0.39	− 0.21	− 0.51*	− 0.08
	Unusual pair	Sync/leader	C	− 0.37	− 0.36	− 0.09	− 0.23	0.24
			CP	− 0.30	− 0.24	− 0.12	− 0.01	0.27
			P	− 0.04	− 0.28	− 0.02	0.10	0.01
		Sync/follower	C	− 0.21	− 0.37	− 0.11	− 0.21	0.13
			CP	− 0.43	− 0.31	− 0.38	− 0.27	0.22
			P	− 0.42	− 0.47	− 0.30	− 0.27	− 0.04

*C* central region, *CP* centro-parietal region, *P* parietal region, *EC* empathic concern, *EQS* emotional intelligence scale, *FS* fantasy, *IRI* Interpersonal Reactivity Index, *PD* personal distress, *PT* perspective taking.

In addition, we performed Bayes factor analyses to evaluate the likelihood of the correlation results. They revealed further evidence of the correlations between the phi components and scores of empathetic questionnaires [BF_10_ = 2.45 for phi 1 on the central region (Sync/Leader, Unusual pair) and PT of IRI; BF_10_ = 2.41 for phi 2 on the central region (Sync/Follower, Usual pair) and Empathy of EQS; BF_10_ = 9.21 for phi 2 on the centro-parietal region (Sync/Follower, Usual pair) and Empathy of EQS; BF_10_ = 2.17 for phi 2 on the centro-parietal region (Sync/Follower, Usual pair) and PT of IRI; BF_10_ = 1.94 for phi 2 on the parietal region (Sync/Follower, Usual pair) and PT of IRI]. Although the FDR correction eliminated all significant correlations, the results of the uncorrected Pearson’s correlations are thought to be still meaningful based on both the Bayesian results and the fact that they coherently reflected characteristics of the phi complex (i.e., sensitivity to empathetic ability and centroparietal dominance).

### Occipital alpha and motor mu rhythms

The phi complex on which we focused in this study was in an alpha-frequency band (i.e., 8–13 Hz). Previous studies have found that the power in this alpha-frequency band could be influenced by some functions of the brain. For example, the alpha powers in the occipital area were influenced by the arousal level (e.g.,^23^), while in the central area they were suppressed by the participants’ own movement and observation of the movement of the other (e.g.,^24^). The same statistical analyses as for the phi complex were applied to the occipital and central alpha (i.e., mu rhythm) to confirm whether these results were specific for the phi complex. In these analyses, the powers in 8–13 Hz in the central (i.e., C3 and C4) and occipital region (i.e., O1 and O2) were calculated as the motor-related and occipital alpha, respectively. In the analyses of the occipital alpha, two participants out of original 16 participants were excluded because of high levels of noise in the occipital electrodes.

The three-way ANOVA with factors of hemisphere (left/ right hemisphere), condition (usual-/ unusual-pair condition) and trial (control/ sync-leader/ sync-follower trials) revealed that there were main effects of hemisphere and trial factors in powers of the central area [*F*(1, 15) = 10.5, *p* = 0.005, η_p_^2^ = 0.41, *F*(2, 30) = 14.1, *p* < 0.001, η_p_^2^ = 0.48, respectively]. The alpha powers were smaller in the left hemisphere than in the right hemisphere. The alpha power was smaller in the sync-follower trial than in in both the control and sync-leader trials [*t*(15) = 6.33, *p* < 0.001, *d* = 1.23, *t*(15) = 3.16, *p* = 0.006, *d* = 0.87, respectively].

The three-way ANOVA was applied to the powers of the occipital area. It revealed a main effect of the trial factor [*F*(2, 26) = 6.99, *p* = 0.004, η_p_^2^ = 0.35]. The alpha power in the sync-follower trial was smaller than that in both the control and sync-leader trials [*t*(13) = 3.86, *p* = 0.006, *d* = 1.21, *t*(13) = 2.86, *p* = 0.040, *d* = 0.91, respectively].

The same correlational analyses as for the phi complex were applied to the occipital and central alpha data. Concretely, in the occipital and central alpha data, we subtracted the powers in the right hemisphere from those in the left hemisphere. Thereafter, we subtracted the value in the control trial from values in the sync trials to remove motor-related neural activities due to wrist movements. These correlational analyses revealed that there were no significant correlations between the alpha powers in the central and occipital area and the empathetic scores of the questionnaires (PT/FS/EC/PD in IRI and Empathy in EQS) (*ps* ≥ 0.13).

## Discussion

The main purpose of this study was to elucidate the neural correlates that underlie synchronized movements in a pair of boat rowers. We compared differences in the phi complex during a simple wrist movement coordination (i.e., neural index of social interaction) between the usual- and unusual-pair conditions. Our results demonstrated that wrist movement frequency was consistent (~ 1 Hz) irrespective of trial type (control, sync-leader, sync-follower trials) or condition (usual- and unusual-pair condition); therefore, motor-related brain activities associated with wrist movements appeared to be similar across these trials and conditions. Moreover, although coordinated movements may rely on the visual perception of the movement of a partner^19^, visual-related brain activities associated with the perception of the partner’s movement appeared to be similar across all trials and conditions (i.e., the visual information projected onto the retina was not different across these trials and conditions). The constant movement frequency across trials and conditions in our study suggested that visual information processing did not differ between sync and control trials in either of the paired conditions.

To clarify which partner led wrist movements, we calculated cross correlations (i.e., a useful index to assess synchronization and time delay between two signals^25^). The zero-lag values obtained by the cross-correlation analysis confirmed that wrist movements in the control trials were not synchronized, whereas a high synchronization was observed in the sync trials. The time lag of the peak of the cross-correlation curve was calculated for each condition. As hypothesized, these results suggest that the wrist movement of a dyadic pair remained synchronized temporally in the sync trial, but not in the control trial. Although instructions for an assignment of the asymmetrical role were provided to participants of a dyad, there was no temporal precedence in wrist movements between wrist movement signals.

One possible explanation for the high phasic synchronization in coordinated movements in the leader–follower relationship is mutual entrainment. Movement of a dyadic pair was unintentionally phase-entrained when a dyad of participants executed a simple rhythmic task with individual tempo^26^. In the synchronization of interlimb rhythmic movement between persons, mutual phase entrainment was observed^27^. Indeed, entrainment often emerges in music, art, and sports^28^.

Strong phasic coordination between two rowers in the leader–follower relationship is a feature of synchronized movement that boat rowers have acquired through practice in double scull rowing. In joint grasping tasks that tested the leader–follower relationship, the followers inhibited automatic resonance using the prediction ability of their partner’s intention^29^ and exhibited mutual adaptability to the current action^30^. In our study, all leaders were asked to move their wrist at a constant frequency. Since the leader’s wrist movements were highly predictable for the follower, they did not significantly precede the follower’s movements. Furthermore, all participants were highly experienced rowers and had acquired skills in synchronizing their movements. Additionally, boat rowers use strategic signalling^31,32^; therefore, leaders may have the ability to provide followers with necessary information by moving their wrist.

We calculated the phi complex to assess neural responses for social interaction in the coordinated wrist movement of boat rowers. According to Tognoli et al. (2007)^20^, augmentation of phi 1 reflects inhibition of the MNS and/or enhancement of intrinsic motor activity, whereas augmentation of phi 2 reflects activation of the MNS, which underpins social interaction. Asymmetrical activities of the phi complex exhibited an inverted-mountain-shape plot in the range of 10 − 13 Hz over the centro-parietal regions. In accordance with Tognoli et al. (2007)^20^, we identified phi 1 in the 10–12 Hz range and phi 2 in the 12–13 Hz range during the sync trials; however, the two peaks were less distinguishable from each other.

We found a stronger augmentation of phi 1 when leading than when following wrist movements, irrespective of conditions. This suggests that the MNS was inhibited when participants acted as leaders during dyadic coordination. This result was consistent with the phase entrainment observed during wrist movements. To act as a leader, the participant had to inhibit the urge to follow their partner’s movements and overcome phase entrainment induced by the in-phase synchronization. Similarly, our correlational analysis implied more obvious suppression of phi 1 in boat rowers scoring higher in PT on the IRI (i.e., higher empathy) during sync-leader trials than during control trials in the unusual-pair condition. This result indicates that MNS inhibition may be critical for the effective synchronization of movements when leading the movements in the unusual-pair condition. However, this is not observed in participants when following movements in the usual-pair condition.

Although phi 1 was modulated by role type in a social interaction, phi 2 did not differ between the control and sync trials. However, correlation analyses showed that there may be correlations between the magnitude of the phi 2 and empathic ability. Boat rowers who had a higher PT score on the IRI produced larger phi 2 magnitudes when synchronizing the movements as a follower than when synchronizing the movement as a leader in the usual-pair condition. These results suggest that the MNS may be more strongly activated in followers than in leaders. A previous study reported that participants with higher PT scores on the IRI showed less social dysfunction and greater social competence than participants with lower PT scores^33^; therefore, rowers with larger phi 2 magnitudes may have greater social skills than rowers with smaller phi 2 magnitudes. The inferior parietal lobule that constructs the MNS is also involved in PT functions^34^. When following a partner’s movements in the double scull, the activation of empathy-specific mirror neurons may be activated alongside the general activation of the MNS.

This assumption is supported by the significant negative correlations between empathy scores on the EQS and phi 2 magnitudes in the usual-pair condition, but not in the unusual-pair condition. The shared attention increased the inter-individual neural activation of the inferior frontal gyrus (i.e., a part of the MNS^22^). The experience of shared attention through daily boat training may impact the MNS. However, it was unlikely that the activation of the inferior frontal gyrus exclusively produced the phi 2 that was distributed over the centro-parietal regions. This may explain why phi 2 did not differ between the usual- and unusual-pair conditions. To reveal generators of the phi 2, a high spatial resolution technique should be adopted in future studies.

To confirm the validity of the analyses of the phi complex, we applied the same statistical analyses as for the phi complex to the alpha powers on the central and occipital regions. The results of the ANOVA on the alpha powers in the central area revealed that the alpha powers were smaller in the sync-follower trial than in the control and sync-leader trials. The alpha power can be an index of the arousal level^23^. In the control and sync-leader trials, participants moved their wrist at their own pace; on the other hand, the effort to adjust their movement to the movement of their partners was required in the sync-follower trial. This difference may explain the difference in the arousal level across trials. This interpretation was consistent with the result of the ANOVA on the alpha power of the occipital region. Moreover, we found that the alpha power was smaller in the left central area than in the right central area. In this study, we asked participants to move their right hand in the wrist movement task. The unilateral hand movement could contribute to the hemispheric difference in the central scalp area, above the primary motor cortex^35,36^. More importantly, in all correlational analyses using the data of the alpha powers on the central and occipital regions, no significant correlations were found. Despite the fact that the phi complex was correlated with the empathy scores of the questionnaire, there was no correlation between the empathy scores and alpha powers of the central and occipital area. These results enhance the validity of the correlation results for phi complex in this study. A positive correlation was found between phi 1 and PT scores in IRI, and negative correlations were found between phi 2 and PT scores in IRI in the correlation analysis of this study. Moreover, negative correlations were found between phi 2 and the empathy score in EQS; it is known that IRI and EQS empathy scores illustrate abilities related to empathy, and that phi 1 and 2 reflect the inhibition and activation of the MNS. The correlations obtained in the present study were what we expected according to an a priori hypothesis including the direction of the correlation. We a priori expected the significant correlations observed before FDR corrections based on previous findings. In addition, no correlation was found for central and occipital alpha powers. Taken together, we believe that the correlations that lost their significance after the FDR corrections may be meaningful.

This study is not without its limitations. One of them was that a subjective part was included in the measurement of phi 1 and 2. In this study, the magnitude of the phi complex was evaluated by measuring the powers in the frequency band, which was defined by visually inspecting the grand-averaged results of central, centro-parietal and parietal electrodes^20^. Although the results of the phi complex in this study were consistent with the functional significance revealed in previous studies, the morphology of the phi 2 remained unclear. Further research on the measurement of the phi complex may be required for it to be used as an index of empathy.

Moreover, the neural mechanism of the phi complex was unclear, which is yet another limitation. It was indicated that the phi complex could be an index for the neural activities for movement coordination. However, the neural mechanism remained unclear. To understand the neural correlates of the coordination of the athletes, future studies using high spatial resolution measurements, such as functional magnetic imaging are warranted.

In conclusion, our phi complex results indicated that the MNS was responsible for social interactions associated with the dyadic coordination of boat rowers. Our findings thereby suggest that precise synchronization may be achieved when the leader inhibits the MNS and the follower activates the MNS. Enhancement of mirror neuron activation in the follower may be especially crucial to achieve sophisticated synchronization, which athletes have acquired through sports training. Strong synchronization may result from a player freely moving and another player following their partner’s movements during dyadic movement synchronization in sporting events. Overall, our results shed light on the neural correlates that underlie effective synchronization in a sports dyad.

## Methods

### Participants

**Figure 5:**
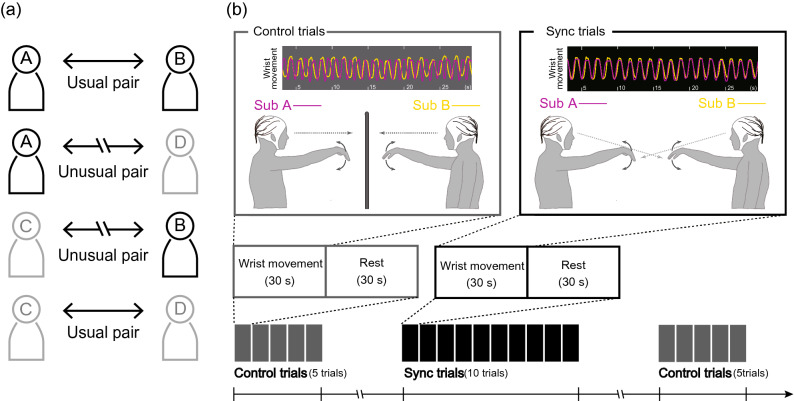
(**a**) Schematic illustration of the pairing. (**b**) Schematic illustration of the wrist movement task. Typical examples of wrist movements in the control and sync conditions.

Nineteen participants aged 18–20 years (*M* ± *SD*: 19.2 ± 0.7) who were members of the Waseda University’s boat team participated in this study. A total of 19 participants, including nine double scull boat pairs, were grouped and participated in the study (four 4-membered groups and one 3-membered group. Each group participated on the same day). Participants were instructed to perform the wrist movement task that required dyadic participants to synchronize wrist movements (see the paragraph on task for details). Participants experienced both two types of conditions: usual- and unusual-pair conditions. All participants in the four 4-membered group experienced both conditions by exchanging the dyadic experimental pair (Fig. 5a). A 3-membered group was composed of a double scull boat pair and a player they had not trained with in the double sculls. Therefore, the participant in the 3-membered group did not complete the two conditions; the data of this participant was excluded. Moreover, two out of the 19 participants were also excluded from further analyses due to EEG artefacts. The remaining 16 participants were aged 18–20 (*M* ± *SD*: 19.2 ± 0.7). Hand preferences of all participants were assessed with the laterality quotient of the Edinburgh Handedness Inventory^37^. All 16 participants showed a preference for the right hand (*M* ± *SD*: 83.8 ± 15.9). This study was approved by the Ethics Committee of Waseda University and carried out in accordance with Ethical Guidelines for Medical and Health Research Involving Human Subjects. Informed consent was obtained from all participants. All participants were paid 3000 Japanese yen (~ $30) for their participation.

### Procedure

Four participants, including two pairs in the double scull, were selected from the boat team and gathered on the same date for participation in the experiment (Fig. 5a). The wrist movement task was performed under the usual- and unusual-pair conditions. Prior to the execution of the wrist movement task, participants were asked to complete the questionnaires. The first questionnaire consisted of the IRI, which is a self-report questionnaire that assesses empathy as a multidimensional construct^33,38^. The IRI is composed of four subscales: (1) PT, (2) fantasy (FS), (3) empathic concern (EC), and (4) personal distress (PD). The PT scale assesses the tendency to view another person’s perspective. The FS scale assesses the tendency to replace oneself as a fictional person. The EC scale assesses the tendency to evoke others-oriented emotion, and the PD scale assesses the tendency to evoke self-oriented feelings of anxiety and fear through the observation of others. The second questionnaire consisted of an emotional intelligence scale (EQS) that assesses emotional intelligence using 65 items. By answering these 65 items, the following nine factors for emotional intelligence were evaluated: (1) self-awareness, (2) self-motivation, (3) self-control, (4) empathy, (5) altruism, (6) interpersonal relationship, (7) situational awareness, (8) leadership, and (9) flexibility. We focused on the empathy scores in order to evaluate empathetic ability, which could be correlated with the activity of the MNS.

### Task

A pair of participants sitting face-to-face performed the wrist movement task (Fig. 5b). During this task, the participants repeatedly flexed and extended their right wrist with or without synchronization of wrist movement. The task included 10 control trials and an equal number of experimental trials. During the control trials, participants were asked to flex and extend their wrist repeatedly, at their own pace, with a cardboard wall between the pair of participants. Therefore, the visual interaction between the pair was eliminated in these trials. During the synchronized trials (sync trial), the participants were asked to observe their partner’s movement and synchronize their wrist movement with that of their partner. In the sync trials, we established a leader–follower relationship between a pair. In the first five experimental trials, one participant was appointed as the “leader”, who was instructed to lead the movement, and the other was the “follower”, who was instructed to move their wrist following the leader’s movement. The roles of leader and follower were reversed in the second five trials. Each task consisted of two blocks with five trials per block. ABBA sequence was applied to reduce the time-course effect (i.e., the order of conditions was alone, pair, and pair and alone condition). Each trial continued for 30 s and was followed by a 30-s rest period. At the beginning of each trial in the alone condition, a visual cue was individually provided to each participant to insert a time lag before the start time of the pair to avoid synchronization of movement.

### Physiological recordings

A dual-EEG system was set up by combining two Brain Amps (Brain Products, Gilching, Germany). This allowed us to record EEGs from a pair of participants without time lag between them. The EEG of each participant was recorded from 28 electrode sites (Fp1, Fp2, F3, F4, Fz, FC1, FC2, FC5, FC6, FCz, C3, C4, Cz, T7, T8, CP3, CP4, CP5, CP6, P3, P4, P7, P8, Pz, PO9, PO10, O1, and O2) of the ActiCap system with 32 channels (Brain Products, Gilching, Germany) according to the 10–20 system montage by Vision Recorder software version 1.20.0601 (Brain Products, Gilching, Germany; https://www.brainproducts.com/productdetails.php?id=21). Electrooculogram (EOG) was recorded in order to monitor vertical and horizontal eye movements. Vertical eye movement was recorded using electrodes that were placed at left infraorbital sites and Fp1. Horizontal eye movement was recorded using a pair of electrodes that were placed at the left and right outer canthi. Electrode impedance was kept below 10 kΩ. The data from all of the channels were filtered [time constant (TC) of 1.6 s to 200 Hz] and digitized with 16-bit resolution at a sampling rate of 1000 Hz. The wrist movement was recorded using flexible goniometers (Biometrics, Ltd., U.K.) at a sampling rate of 1000 Hz.

### Data analysis

The EEG and behavioural data during the execution of the wrist movement task was segmented into epochs of 26.5 s (both time periods of 3 s after the onset and 0.5 s prior to the end of the task were eliminated to reduce contamination of the psychophysiological data).

The offline processing of EEG data was performed using Brain Vision Analyzer 2 version 2.0.4.368 (Brain Products, Gilching, Germany; https://www.brainproducts.com/productdetails.php?id=17). EEG data was collected and assessed during the 30-s wrist movement in each condition. The average-referenced EEG data were band-pass filtered with a TC of 0.3 s–20 Hz cut-off (roll-off: 24 dB/octave). A 50 Hz notch filter was applied to the EEGs to remove power supply frequency noise. After segmentation by condition, ocular-movement artefacts were removed from the EEG data using an algorithm described by Gratton, Coles, & Donchin (1983)^39^. All epochs were averaged after calculating the power spectra using a fast Fourier transform with a Hamming window. In the current study, a high-resolution spectral analysis was applied according to Tognoli et al. (2007)^20^.

In this study, we calculated the phi complex in each condition for each participant. According to Tognoli et al. (2007)^20^, the phi complex is assessed by subtracting the power in the right hemisphere from the power in the left hemisphere over the frontal, central, centro-parietal, and parietal regions. By visual inspection of the grand-averaged results, the mean powers of 10 − 12 and 12 − 13 Hz were scored as phi 1 and phi 2, respectively.

The wrist movement data, which was acquired as voltage data, were first converted to the angle of wrist flexion and extension using the calibration data. The angle data of the wrist movement were normalized to z-scores. Wrist movement frequency was identified by calculating the first peak latency from the results of the auto correlation analysis of a wrist movement signal. To assess the synchronization of dyadic coordination, a cross correlation was applied. Auto and cross correlations were thereafter calculated using formulas (1) and (2), respectively,

1 $${R}_{xx}\left(\uptau \right)=\frac{1}{N}\sum_{t}^{N+t-1}\frac{\left(x\left(t+\tau \right)-\stackrel{-}{x}\right)\left(x(t\right)-\stackrel{-}{x})}{{\sigma }_{x}{\sigma }_{x}}$$

2 $${R}_{xy}\left(\uptau \right)=\frac{1}{N}\sum_{t}^{N+t-1}\frac{\left(x\left(t+\tau \right)-\stackrel{-}{x}\right)\left(y(t\right)-\stackrel{-}{y})}{{\sigma }_{x}{\sigma }_{y}}$$

where $x$ represents the signal of the wrist movement for one participant, and $y$ represents the signal of the wrist movement for the other participant. $\stackrel{-}{x}$ and $\stackrel{-}{y}$ represent the mean values of the signals, ${\sigma }_{x}$ and ${\sigma }_{y}$ represent the standard deviations of the signals, and $N$ is the total sampling points of the signals (i.e., 26.5 s × 1000 Hz). $\uptau$ represents a time lag between two signals in the analysis. Auto- and cross-correlation analyses were applied to the wrist movement data in each trial (Fig. 1a). The individual results of the auto- and cross-correlation analyses were calculated by averaging all trials of each condition.

### Statistical analyses

Statistical analyses were conducted using IBM SPSS Statistics version 22 (IBM Corp., NY, USA; https://www.ibm.com/products/spss-statistics). Power values of phi 1 and phi 2 were analysed by repeated-measures analysis of variance (ANOVA) using trial (sync-leader/sync-follower/control trials), condition (usual-/ unusual-pair condition), and region (frontal/central/centroparietal/parietal area) as the within-subject factors. When the Mauchly’s test revealed a violation of the assumption of sphericity, ε values were reported in addition to the *F* and *p* values. Two-tailed paired *t*-tests were conducted for post-hoc comparisons of the results of ANOVAs. In the post-hoc comparisons, Bonferroni correction was used when necessary. Before applying a parametric analysis, the normality of the distribution of the data was tested by the Shapiro–Wilk test with multiple testing correction by the FDR approach. Given that the *F*-test is robust in terms of Type I error (e.g.,^40^), the results of ANOVAs may be meaningful. When the Shapiro–Wilk test revealed a violation of the normality, both the results of parametric and non-parametric analysis were described. In the post-hoc comparisons for the data with the non-normal distribution, the Wilcoxon signed-rank test was used. Pearson’s correlation analyses were performed to assess the relationships of the phi complex magnitudes with each scale of the IRI and the empathy score of the EQS. The FDR approach was used as the alpha adjustment method for multiple correlation correction^41,42^. The adjusted alpha was calculated using 12 correlations [3 regions (central/centroparietal/parietal area) × 2 conditions (usual-/ unusual-pair condition) × 2 trials (sync-leader/sync-follower trials)], separately for each item of the questionnaires and for each component of the phi complex. In addition, we performed Bayesian analyses to provide further evidence for the correlations by using the JASP software version 0.14.1 (JASP Team; https://jasp-stats.org/). In the Bayesian correlations, we calculated the Bayes factor (BF) representing the likelihood of the null and alternative hypothesis based on the acquired data^43,44^. We reported BF_10_ as an index of the likelihood that the alternative hypothesis might prevail the null hypothesis (e.g., “BF_10_ = 2” indicated that the likelihood of the alternative hypothesis was twice larger than the null hypothesis).

The wrist movement frequencies were analysed by a two-way ANOVA using trial and condition as the within-subject factors. Moreover, the cross-correlation between two signals evaluated the synchronization of wrist movements in a dyadic pair. The peak time of the cross-correlation curve in each condition of the sync trial was compared with 0 ms using a one-sample *t* test.

## Data Availability

The data used for this study are available upon request from the corresponding author.
